# Relationship between circulating miR-132 and non-alcoholic fatty liver disease in a Chinese population

**DOI:** 10.1186/s41065-020-00136-y

**Published:** 2020-05-22

**Authors:** Yicen Zong, Jing Yan, Li Jin, Bo Xu, Zhen He, Rong Zhang, Cheng Hu, Weiping Jia

**Affiliations:** Shanghai Diabetes Institute, Shanghai Key Laboratory of Diabetes Mellitus, Shanghai Clinical Center for Diabetes, Department of Endocrinology and Metabolism, Shanghai Jiao Tong University Affiliated Sixth People’s Hospital, 600 Yishan Road, Shanghai, 200233 China

**Keywords:** NAFLD, T2DM, miR-132, TG, ALT

## Abstract

**Background:**

Non-invasive diagnostic markers are of great importance for early screening nonalcoholic fatty liver disease (NAFLD). MicroRNAs (miRNAs) play significant roles in many metabolic disease, including NAFLD. Therefore, this study focusd on a Chinese population to explore the possible correlation between circulating miR-132 and NAFLD.

**Results:**

Serum miR-132 was positively associated with NAFLD in non-type 2 diabetes mellitus (T2DM) groups by logistic regression (OR = 3.082 [1.057, 8.988], *P* = 0.039) after adjusting age, sex, and body mass index (BMI). Additionally, in non-T2DM subgroup, after adjusting age, sex, bmi, serum miR-132 was significantly associated with ALT (β ± SE = 0.005 ± 0.002, *P* = 0.018), TG (β ± SE = 0.072 ± 0.029, *P* = 0.015), FPG (β ± SE = 0.123 ± 0.058, *P* = 0.036), γ-GT (β ± SE = 0.002 ± 0.001, *P* = 0.047), apoE (β ± SE = 0.038 ± 0.002, *P* = 0.017) .

**Conclusions:**

Serum miR-132 was found to be associated with NAFLD risk in a Chinese cross-section study. This finding provides a prospective research direction for early screening and diagnosing NAFLD.

## Background

With development of social economic and transition of lifestyle, non-alcoholic fatty liver disease (NAFLD) has evolved into a serious threat to human health [1]. The pathological development of NAFLD is a process of continuous aggravation and deterioration, ranging from initial hepatic steatosis to steatohepatitis (NASH), then fibrosis, further to cirrhosis, and eventually result in hepatocellular carcinoma (HCC) [2]. Over the past few decades, NAFLD has replaced viral hepatitis as the highest incidence chronic liver disease, affecting almost 25% of adults globally, due to high calorie diet and a lack of exercise [3].

At present, liver biopsy has been considered as the most convincing tool for diagnosing NAFLD [4], but the potential risks of its invasiveness make it unsuitable for screening at-risk populations. In addition, although diagnostic accuracy is satisfactory, the high cost and complexity of the Magnetic Resonance Imaging (MRI) procedure have limited its clinical application for NAFLD diagnosis [5]. Recently, benefited from their convenience in clinical auxiliary diagnosis, circulating biomarkers have become a convenient and reliable tool for the detection and prediction of NAFLD.

MicroRNAs (miRNAs) are a class of non-coding single-stranded RNAs with a length of about 22 nt that regulate gene expression in post-transcriptional stage in plants and animals [6]. MiRNA-mediated gene silencing can alter many biological functions, such as energy metabolism, tissue inflammation, and liver dysfunction [7]. Mature miRNAs may enter the blood through passive releasing or by binding to microvesicles or lipoprotein complexes. Thus, they are hard to be degraded by RNases in the serum so that enables their stability [8, 9]. These facts suggest that disease-related miRNAs in serum and plasma may be regarded as potential circulating biomarkers. In the progression of NAFLD, various miRNAs in the liver can be released into the bloodstream. Thus, identification of changes in circulating miRNAs driven by disease progression have been applied to detection and diagnosis of NAFLD [10].

The miR-132 gene is derived from an intron region located on mice chromosome 11 or humans chromosome 17 [11, 12]. It regulates multiple biological functions, such as nutrition metabolism [13], cell proliferation [12], epigenetic modification [14], and inflammatory infiltration [15]. Several in vivo studies showed that miR-132 was correlated with hepatic steatosis, hyperlipidemia, and glucose metabolism [16–18] by targeting its related genes, such as *Sirt1*. Hanin et al. found that liver miR-132 levels increased significantly in a white NAFLD population compared to controls, and anti-miR-132 therapy could reverse hyperlipemia in high-fat-diet mouse models [19]. Considering a lack of miR-132 investigations of the disease and relative metabolic characters in Chinese population, our study aimed to elucidate the clinical relevance of miR-132 in a Chinese cohort of NAFLD subjects.

## Results

### Clinical parameters of individuals

Table 1 presents the characteristics of individuals in this study. No difference was found in age comparing subjects whether have NAFLD or not, no matter which group the individuals come from. Similar situations exist in sex as well as body mass index (BMI). Consistent with diagnosis, NAFLD groups showed obviously higher aspartate aminotransferase (AST), alanine aminotransferase (ALT), γ-glutamyl transpeptidase (γ-GT), triglyceride (TG), fasting plasma glucose (FPG), 2-h plasma glucose (2hPG), and apolipoprotein E (APOE) than the subjects without NAFLD in non-T2DM group (*P* <  0.05). Instead, HDL-C levels were relatively lower in individuals with NAFLD than without, from the *p* value of 0.002 in t-test. Actually, there is a little bit different in the T2DM group. NAFLD subjects still had higher ALT, AST, γ-GT, TG inspecting values, while total cholesterol (TC) along with low-density lipoprotein cholesterol (LDL-C) levels showed much higher compared with no NAFLD subjects (with a *p* value< 0.05).

**Table 1:** Anthropometric parameters and clinical characteristics of participants

	Non-diabetes group		*P* values	Diabetic group		*P* value
	Control (*N* = 67)	NAFLD (*N* = 73)		Control (*N* = 68)	NAFLD (*N* = 66)	
**Age (years)**	57.87 ± 6.84	57.28 ± 6.66	0.605	57.74 ± 6.83	57.21 ± 6.46	0.647
**Male/female (n)**	47/20	50/23	0.832	46/22	46/20	0.798
**BMI (kg/m**^**2**^**)**	24.39 ± 1.96	24.89 ± 2.14	0.156	57.74 ± 6.83	57.21 ± 6.46	0.647
**FPG (mmol/L)**	5.56 ± 0.47	5.82 ± 0.50	0.002	7.22 ± 1.29	7.15 ± 1.31	0.757
**2 h PG (mmol/L)**	7.04 ± 1.61	8.03 ± 1.64	< 0.001	12.95 ± 3.69	12.85 ± 3.31	0.87
**HbA1C (%)**	5.58 ± 0.31	5.59 ± 0.40	0.778	6.17 ± 1.12	6.45 ± 0.83	0.108
**ALT (U/L)**	20.58 ± 5.40	33.52 ± 13.84	< 0.001	16.59 ± 6.96	23.14 ± 10.96	< 0.001
**AST (U/L)**	22.81 ± 5.17	27.51 ± 9.03	< 0.001	21.09 ± 5.25	23.03 ± 5.54	0.039
**GGT (U/L)**	22.85 ± 8.00	47.56 ± 28.84	< 0.001	22.76 ± 9.04	35.98 ± 23.31	< 0.001
**TG (mmol/L)**	1.25 ± 0.60	2.02 ± 1.11	< 0.001	1.38 ± 0.77	1.98 ± 1.13	< 0.001
**TC (mmol/L)**	5.19 ± 0.93	5.35 ± 1.02	0.350	4.97 ± 0.90	5.34 ± 0.93	0.021
**LDL-C (mmol/L)**	3.24 ± 0.77	3.36 ± 0.84	0.357	3.03 ± 0.75	3.36 ± 0.75	0.012
**HDL-C(mmol/L)**	1.36 ± 0.29	1.22 ± 0.24	0.002	1.28 ± 0.26	1.24 ± 0.29	0.349
**APOA (mmol/L)**	1.39 ± 0.28	1.4 ± 0.26	0.783	1.45 ± 0.30	1.38 ± 0.33	0.179
**APOB (mmol/L)**	0.89 ± 0.17	0.93 ± 0.17	0.093	0.87 ± 0.15	0.94 ± 0.15	0.068
**APOE (mmol/L)**	4.26 ± 1.56	5.07 ± 2.01	0.009	4.34 ± 1.77	4.85 ± 1.66	0.090
**FFA (umol/L)**	503.16 ± 185.25	528.46 ± 152.50	0.382	647.09 ± 249.14	640.25 ± 244.14	0.874

Data are shown as mean ± standard deviation. *P* value indicates the comparison of non-alcoholic fatty liver disease (NAFLD) and control subjects

*Abbreviations*: *2hPG* 2-h plasma glucose, *FPG* Fasting plasma glucose, *ALT* Aspartate aminotransferase, *AST* Alanine aminotransferase, *ALT* Alanine aminotransferase, *TG* Triglyceride, *TC* Total cholesterol, *APOE* Apolipoprotein E, *APOA* Apolipoprotein A, *APOA* Apolipoprotein A, *HDL-C* High-density lipoprotein cholesterol, *LDL-C* Low-density lipoprotein cholesterol, *FFA* Free fatty acids

### Association between serum miR-132 and NAFLD risk

Exploration of relevance between circulating miR-132 and NAFLD risk in T2DM and non-T2DM group were performed separately. As shown in Table 2, logistic regression revealed that circulating miR-132 levels were nominally correlated with NAFLD risk in non-T2DM group after adjusting sex, age, BMI (Odds ratio [OR] = 3.082, 95% CI [1.057, 8.988], *P* = 0.039). However, the same association was not observed in individuals with T2DM (OR = 0.793, 95% CI [0.349, 1.802], *P* = 0.580). Further, in analysis of overall individuals, although circulating miR-132 levels increased as the risk of NAFLD increased, it had no statistical significance (*P* = 0.378).

**Table 2:** Association between miR-132 and NAFLD in different groups by logistic regression after adjusting for age, sex, and BMI

Groups	OR (95%CI)	*P* value
Non-diabetic group (*N* = 140)	3.082 (1.057, 8.988)	0.0392^*^
Diabetic group (*N* = 134)	0.793 (0.349, 1.802)	0.5800
Overall^1^ (*N* = 274)	1.332 (0.704, 2.518)	0.3782
Overall^2^ (*N* = 274)	0.751 (0.397, 1.421)	0.3790

The odds ratio (OR) and 95% confidence interval for the association of miR-132 and non-alcoholic fatty liver disease (NAFLD) in non-diabetic and diabetic groups after adjustment for age, sex, and body mass index

Overall^1^ means logistic regression model preformed in whole individuals that adjusted age, sex, bmi

Overall^2^ means logistic regression model performed in whole individuals that adjusted age, sex, bmi and diabetes status

* means *P*<0.05 and has statistic significance

### Association between serum miR-132 and quantitative traits related to NAFLD

**Fig. 1:**
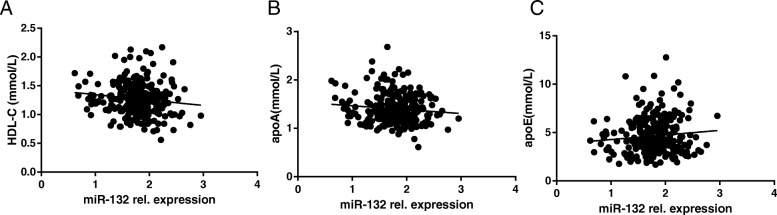
Correlations of serum miR-132 with biochemical indicators in all individuals: **a** HDL-C; **b** APOA; **c** APOE. A simple correlation analysis was adopted. miR-132 rel.expression, the relative expression level of miR-132 normalized against the level of miR-191

We further analyzed the effects of miR-132 on clinical characteristics in all individuals. Pearson’s correlation showed that miR-132 was correlated with HDL-C levels (*r* = − 0.130, *P* = 0.015), apoE (*r* = 0.100, *P* = 0.049), and apoA (*r* = − 0.103, *P* = 0.044), The outcomes were shown in Table 3 and Fig. 1. Nevertheless, there is no obvious correlations between miR-132 and indicators related to liver function, AST, ALT, and γ-GT, for instance. Furthermore, multiple linear regression indicated that miR-132 was still nominally associated with HDL-C and apoE (β ± SE = − 0.176 ± 0.085, *P* = 0.040 for HDL-C; β ± SE = 0.024 ± 0.013, *P* = 0.049 for apoE, respectively) after adjusting sex, age, and BMI.

**Table 3:** Simple correlations of log-transformed serum miR-132 with clinical parameters

	*r*	*P* values
**Age (years)**	− 0.034	0.288
**BMI (kg/m**^**2**^**)**	0.018	0.386
**FPG (mmol/L)**	0.0508	0.204
**HbA1C (%)**	−0.047	0.218
**ALT (U/L)**	0.078	0.099
**AST (U/L)**	0.031	0.302
**γ-GT (U/L)**	0.069	0.254
**TG (mmol/L)**	0.087	0.072
**TC (mmol/L)**	−0.090	0.069
**FFA (umol/L)**	−0.088	0.074
**APOA (mmol/L)**	−0.103	0.044*
**APOB (mmol/L)**	−0.012	0.431
**APOE (mmol/L)**	0.100	0.049*
**ALP (U/L)**	−0.002	0.489
**HOMA-IR**	0.074	0.112
**2hPG (mmol/L)**	−0.006	0.970
**LDL-C (mmol/L)**	−0.084	0.084
**HDL-C (mmol/L)**	−0.130	0.016*

The simple correlation coefficient (r) for association between log10-transformed serum miR-132 with clinical parameters. *P* value indicates the relationship of clinical traits and miR-132

* means *P*<0.05 and has statistic significance

**Fig. 2:**
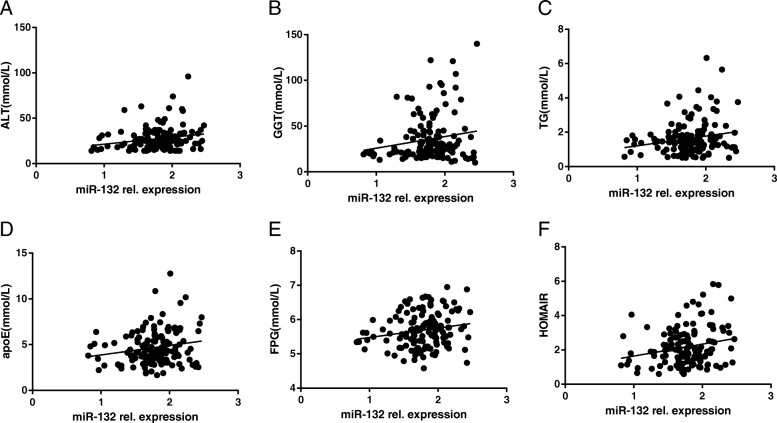
Correlations of serum miR-132 with biochemical indicators in non-T2DM group: **a** ALT; **b** γ-GT; **c** TG; **d** APOE; **e** FPG; **f** HOMA-IR. A simple correlation analysis was adopted. miR-132 rel.expression, the relative expression level of miR-132 normalized against the level of miR-191

The relationship between serum miR-132 and metabolic markers was further analyzed in the non-T2DM subgroup. As shown in Table 4 and Fig. 2, serum miR-132 was positive correlated with ALT, TG, FPG, γ-GT, APOE, HOMA-IR. Moreover, after adjusting for age, sex, bmi, the following multiple linear regression demonstrated that serum miR-132 was still significantly associated with ALT (β ± SE = 0.005 ± 0.002, *P* = 0.018), TG (β ± SE = 0.072 ± 0.029, *P* = 0.015), FPG (β ± SE = 0.123 ± 0.058, *P* = 0.036), γ-GT (β ± SE = 0.002 ± 0.001, *P* = 0.047), APOE (β ± SE = 0.038 ± 0.002, *P* = 0.017). To further evaluate which variables were associated with miR-132 independently, we carried out a multiple stepwise regression analysis by taking into account of sex, age, BMI, ALT, TG, FPG, and APOE, which showed that ALT was independently and dramatically associated with serum miR-132 (β ± SE = 0.005 ± 0.002, *P* = 0.033).

**Table 4:** Simple correlations of log-transformed serum miR-132 with clinical parameters in non-T2DM groups

	*r*	*P* values
**Age (years)**	−0.074	0.386
**BMI (kg/m**^**2**^**)**	0.010	0.910
**FPG (mmol/L)**	0.184	0.029*
**HbA1C (%)**	0.138	0.105
**ALT (U/L)**	0.206	0.015*
**AST (U/L)**	0.142	0.094
**γ-GT (U/L)**	0.173	0.041*
**TG (mmol/L)**	0.195	0.021*
**TC (mmol/L)**	−0.007	0.931
**FFA (umol/L)**	−0.085	0.321
**APOA (mmol/L)**	−0.040	0.641
**APOB (mmol/L)**	0.058	0.496
**APOE (mmol/L)**	0.187	0.027*
**ALP (U/L)**	−0.010	0.911
**HOMA-IR**	0.213	0.010*
**2hPG (mmol/L)**	0.066	0.442
**LDL-C (mmol/L)**	−0.021	0.806
**HDL-C (mmol/L)**	−0.123	0.146

The simple correlation coefficient (r) for association between log10-transformed serum miR-132 with clinical parameters. *P* value indicates the relationship of clinical traits and miR-132

* means *P*<0.05 and has statistic significance

## Discussion

In the study, we investigated the associations of miR-132 with NAFLD risk in a Chinese population and found that serum miR-132 were positively significantly associated with NAFLD. Because the associations between miR-132 and NAFLD have been evaluated only in the liver of white population [19], we decided to carry out this study to investigate whether serum miR-132 levels related to NAFLD risk in a Chinese population.

Previous relative studies have shown that miR-132 gene participated in regulating many liver metabolic functions, such as hepatic steatosis and insulin resistance [20]. Hanin et al. previously reported that miR-132 was highly expressed in NAFLD populations compared with healthy controls [19]. Moreover, Estep et al. demonstrated that with the progression of NAFLD, non-alcoholic steatohepatitis (NASH) patients showed higher expression of miR-132 than patients with NAFLD [21]. Transgenic mice that overexpressed miR-132 exhibited a more severe fatty liver phenotype when fed with high fat, as well as higher LDL/VLDL, liver triglycerides, and increased lipid accumulation-related transcripts, including Pten, FoxO3, and P300 [19]. Consistent with these reports, we demonstrated that increased serum miR-132 was correlated with NAFLD risk in non-diabetic groups in this cross-sectional population study. However, the same association can’t be observed in the T2DM groups, likely due to the influence of T2DM on lipid metabolism. T2DM is an important risk factor for NAFLD. People with T2DM have more fat accumulation, which produces more free fatty acids through de novo lipogenesis, and excess FFA plays a pivotal role in the process of hepatic triglycerides storage [22–24].

Moreover, we investigated the associations of miR-132 with multiple clinical characteristics. Our study found a positive correlation of miR-132 with APOE as well as a negative correlation with HDL-C in all individuals. The literatures reported that in patients with NAFLD the concentration of HDL-C was significantly reduced, whereas APOE, APOB, APOC II and III increased [25]. APOE is known to be mainly synthesized in the liver and transported in CM, VLDL, LDL, and partial HDL. In vitro study showed that a large amount of triglyceride deposition appeared in hepatocytes with increased extracellular APOE levels [26], which is consistent with our findings.

Meanwhile, because of the significant impact of T2DM on NAFLD, we analyzed the relationship between serum miR-132 and metabolic traits in the non-T2DM subgroup. We found a significant correlation between miR-132 and TG levels. Endogenous triglycerides are mainly dependent on intrahepatic synthesis, when the amount of intrahepatic synthesis exceeds its transport capacity, excessive deposition of triglycerides in hepatocytes induces fatty liver, while serum TG level reflects the degree of steatosis of NAFLD [27]. Consistent with reports mentioned above, miR-132 positively correlated with serum TG, this result further illustrated the correlation between miR-132 and NAFLD. As a manifestation of metabolic syndrome in the liver, NAFLD often companies with insulin resistance. Then our study found serum miR-132 positively correlated with FPG and marginally positively associated with HOMA-IR. Our results also indicated serum miR-132 levels were positively correlated with serum ALT, γ-GT, after adjusting age, sex and bmi (all *P*<0.05). Serum ALT is mainly found in the liver, heart and skeletal muscle, and is a common enzymatic indicator reflecting liver cell damage. γ-GT is another important enzyme in the liver, which is mainly involved in glutathione synthesis and catabolism, amino acid transport and detoxification. These increasing liver enzymes are considered to be the most significant biomarkers of liver injury in serum [28]. Correlation analysis indicated positive correlation trend between AST and serum miR-132, In fact, comparing with ALT, AST represents more severe liver damage, a large amount of patients with liver fatty changes had normal AST levels [29]. Therefore, our results may indicate that miR-132 was involved in lipid metabolism and may reflect the liver damage to some extent.

We acknowledged limitations of this study. First, we just demonstrated the association between miR-132 and NAFLD risk in a retrospective study, a predictive effect of miR-132 on NAFLD progression is lacking. Thus, further prospective population studies are needed to explore whether miR-132 could induce NAFLD. Second, the population sample size of the study is somewhat small and lack of liver biopsy samples. It remains a doubt whether miR-132 induces fatty liver or just a feedback change in pathologic process of NAFLD. A large amount of liver biopsy samples is needed to clarify the causal mechanisms involved. Because we did not use biopsy to diagnosis NAFLD which was the ‘golden standard’ of defining procession of NAFLD, we can’t evaluate the role of miR-132 in different stages of NAFLD.

## Conclusion

In conclusion, this study indicates an association between serum miR-132 levels, NAFLD, APOE, and HDL-C levels in a Chinese population. In the future, functional studies will be conducted to define the relationship and underlying mechanism of these findings.

## Methods

This study was a cross-sectional study of 274 people in China to analyze the association between serum mir-132 levels and the risk of NAFLD.

### Individuals

**Fig. 3:**
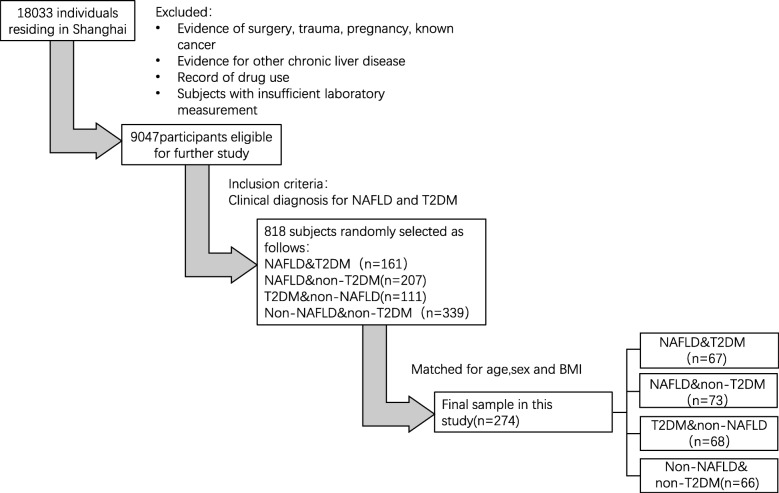
Flow diagram of patient selection. BMI, body mass index; NAFLD, non-alcoholic fatty liver disease; T2DM, type 2 diabetes mellitus

The individuals enrolled in the study were selected from a prospective population-study (*n* = 18,033) in Shanghai designed to explore the occurrence of a series of metabolic diseases. Subjects would be excluded if past-history containing cancer, surgery, drug administration, mental, in pregnancy, or exist other liver diseases. Actually, a total of 5 communities in Shanghai were followed up in this study, one community population (*n* = 850) was randomly selected from these populations, and subjects with definite diagnosis of NAFLD and T2DM were finally enrolled in our further analysis (*n* = 818). By checking the previous diagnosis about NAFLD or/and T2DM, and further matching age, sex, BMI, eventually 274 participants were recruited in this study: 67 subjects with both NAFLD and T2DM, 73 subjects with only NAFLD, 68 subjects with only T2DM, and 66 healthy controls. The whole selection criteria for participants is shown in Fig. 3.

NAFLD was diagnosed according to the Asia-Pacific Working Party definition [30]. All enrolled NAFLD patients are proved by ultrasonography, furthermore those who had the clinical history of alcohol consumption, hepatitis, drug-induced liver disease were also excluded. T2DM was defined by following the rule of 1999 World Health Organization definition [31]. Subjects who had past-history of T2DM or taken anti-diabetic drugs were excluded. Before enrollment, all participants have submitted informed consent voluntary. This study has been warranted by the Ethical Committee of Shanghai Jiao Tong University Affiliated Sixth People’s Hospital.

### Clinical and laboratory assessment

All participants filled out a standard medical questionnaire to exclude diseases or medical history interfered outcomes of this study. The staff measured and recorded the height, weight and waist circumference of all subjects. All individuals’ blood samples were collected at 0 min and 120 min after a oral glucose tolerance test (75 g glucose). HbA1c levels were determined by HPLC using an HLC-723 g7 automated glycoprotein analyzer (Tosoh, Tokyo, Japan). ALT, γ-GT and lipid profiles consisted of triglyceride, total cholesterol as well as various lipoprotein were measured in type 7600 Automated Analyser (Hitachi, Tokyo, Japan).

### RNA isolation and miRNA quantitation

Whole blood samples were taken and placed in the anticoagulant tubes and immediately transfered to cryogenic centrifuge, set the speed to 3000 g for 8 min. After centrifugation, carefully aspirate the upper clarified plasma and store it in a refrigerator at − 80 °C for preservation and further experiments. According to the instructions of miRNeasy Kit (QIAGEN, Valencia, CA, USA), 3.5 μL cel-miR-39 Spike-In Control were needed to add into 200-μL serum samples for ensuring the extraction efficiency of total RNA. After extraction, reverse-transcribing total RNA to cDNA using miScript II RT Kit (QIAGEN). Subsequently, in accordance with the miScript SYBR® Green PCR Kit, the aimed miRNA levels were quantified by 7900HT Fast Real-Time PCR System (Applied Biosystems, Foster City, CA, USA). MiR-191 was quantified for normalization of miR-132 expression due to its stability and detectable Ct using the GeNorm and NormFinder software. The Primer sequences of selected internal reference and target miRNA are showed in Table 1. Finally, among 276 serum RNA samples, 274 samples with valid value of miR-132 were detected.

### Statistical analysis

All continuous variables are shown as mean ± standard deviation (SD). Before analysis, all skewed distribution variables were logarithmically converted into approximate normal distribution. Statistical comparisons of clinical parameters from two groups were conducted by means of Student’s unpaired t-test. After adjusting age, sex, and BMI as confounding factors, the association between miR-132 and NAFLD in T2DM/non-T2DM groups was performed separately with logistic regression. The Linear regression and Simple correlation were performed to explore relevance in serum miR-132 and clinical characteristics. In addition, the independent relationship between the characteristics and circulating miR-132 levels was evaluated by multiple stepwise regression analysis. GraphPad Prism (version 6.02) was used to plot histograms. A two-tailed *P* value < 0.05 indicated statistically significant. All the data analysis steps dependent on SAS 9.3 (SAS Institute, Cary, NC, USA).

## Supplementary information

**Additional file 1: Table S1.** Primer assays for individual miRNAs.

## Data Availability

The datasets used and analysed during the current study are available from the corresponding author on reasonable request.

## Abbreviations

apoA: ApolipoproteinA
apoB: ApolipoproteinB
apoE: ApolipoproteinE
ALT: Alanine aminotransferase
AST: Aspartate aminotransferase
BMI: Body mass index
EDTA: Ethylenediamine tetraacetic acid
HDL-C: High density lipoprotein
LDL: Low density lipoprotein
miRNA: MicroRNA
NAFLD: Non-alcholic fatty liver disease
OGTT: Oral glucose torlence test
TC: Total cholesterol
TG: Triglycerides
T2DM: Type2 diabetes mellitus
γ-GT: γ-glutamyl transpeptidase
