# Supplementary material for: Relationship between circulating miR-132 and non-alcoholic fatty liver disease in a Chinese population
Source: Hereditas. 2020 May 22;157:22. doi: 10.1186/s41065-020-00136-y (PMC7245036; doi:10.1186/s41065-020-00136-y)
Supplement: Supplementary file 1 — Additional file 1: Table S1. Primer assays for individual miRNAs. [file 41065_2020_136_MOESM1_ESM.docx]

**TABLE S1** Primer assays for individual miRNAs

| **Mature miRNA** | **Qiagen ID** | **Sequence (5’→3’)** |
| --- | --- | --- |
| hsa-miR-132-3p | MS00003458 | UAACAGUCUACAGCCAUGGUCG |
| hsa-miR-191-5p | MS00003682 | CAACGGAAUCCCAAAAGCAGGCUG |

The QIAGEN ID indicates the specific miRNA primer assay in QIAGEN (Valencia, CA, USA).
